# Implementing a genomic data management system using iRODS in the Wellcome Trust Sanger Institute

**DOI:** 10.1186/1471-2105-12-361

**Published:** 2011-09-09

**Authors:** Gen-Tao Chiang, Peter Clapham, Guoying Qi, Kevin Sale, Guy Coates

**Affiliations:** 1 Wellcome Trust Sanger Institute, Informatics System Group, Wellcome Trust Genome Campus, Hinxton, CB10 1SA, UK; 2 Wellcome Trust Sanger Institute, New Sequencing Technologies, Wellcome Trust Genome Campus, Hinxton, CB10 1SA, UK; 3 Wellcome Trust Sanger Institute, Infrastructure Management Team, Wellcome Trust Genome Campus, Hinxton, CB10 1SA, UK

## Abstract

**Background:**

Increasingly large amounts of DNA sequencing data are being generated within the Wellcome Trust Sanger Institute (WTSI). The traditional file system struggles to handle these increasing amounts of sequence data. A good data management system therefore needs to be implemented and integrated into the current WTSI infrastructure. Such a system enables good management of the IT infrastructure of the sequencing pipeline and allows biologists to track their data.

**Results:**

We have chosen a data grid system, iRODS (Rule-Oriented Data management systems), to act as the data management system for the WTSI. iRODS provides a rule-based system management approach which makes data replication much easier and provides extra data protection. Unlike the metadata provided by traditional file systems, the metadata system of iRODS is comprehensive and allows users to customize their own application level metadata. Users and IT experts in the WTSI can then query the metadata to find and track data.

The aim of this paper is to describe how we designed and used (from both system and user viewpoints) iRODS as a data management system. Details are given about the problems faced and the solutions found when iRODS was implemented. A simple use case describing how users within the WTSI use iRODS is also introduced.

**Conclusions:**

iRODS has been implemented and works as the production system for the sequencing pipeline of the WTSI. Both biologists and IT experts can now track and manage data, which could not previously be achieved. This novel approach allows biologists to define their own metadata and query the genomic data using those metadata.

## Background

The Wellcome Trust Sanger institute (WTSI) is one of the world's major sequencing centres. It was the largest single contributor to the Human Genome Project (HGP). Although the cost of DNA sequencing is reducing and even a small lab is now able to buy its own sequencers, the WTSI is still the major contributor and produces about 8% of the world's sequencing data.

In 2001, in the era of the HGP, DNA sequencing technology used a capillary-based approach. Each sequencer produced about 115 kbp (thousand base pairs) per day [1]. Since 2005, several next generation sequencers (NGS) based on the massively parallel sequencing approach have been released onto the market. The WTSI has migrated from capillary sequencers to Roche 454 and Illumina GAII sequencers, which can produce 10^13 ^kbp per day from each machine [1].

Some large projects have been launched with the benefit of NGS technologies. For example, the 1000 Genomes Project is an international collaboration project launched in 2008. The project aims to develop a very detailed catalogue of human genetic variations in different human populations. The total storage requirement for the project is about 500 TB. By way of comparison, the data of the 1000 Genomes Project will be about 60 times more than the original HGP [2].

In addition to the 1000 Genomes Project, the WTSI launched the UK10K project in July 2010. UK10K aims to better understand the link between low-frequency and rare genetic changes and human disease caused by harmful changes to the proteins the body makes. The data which UK10K generates will provide a genotype/phenotype resource that will be an order of magnitude deeper than the genetic-only 1000 Genomes Project data set for Europe [3]

However, the data deluge never ends and new sequencing technology keeps evolving. Since October 2010, the WTSI has migrated to Illumina HiSeq 2000, which produces about 5 times more data than Illumina GAII. The storage and computation requirements of HiSeq 2000 have brought the WTSI to a point where traditional data management methods (flat files, databases) [4] have become saturated. A replacement data/metadata management and file tracking system is therefore required. Without a novel data management approach, users will spend most of their time just looking for data, instead of using these data to carry out their research.

Although some advanced file systems (such as Lustre [5], GPFS [6]) are currently available, these were not designed to provide user metadata. The metadata of these file systems merely provide system level metadata; for example, the size of the file, the group or owner, or the creation or modification time. In real usage users need more information, such as project name and security or access classification. Ideally, that information should be inserted when files are staged into the file systems. Users can then access these data by querying the information provided by the user metadata. iRODS is designed to manage multiple file systems transparently and provides features which help to manage user metadata.

Even if the storage capacity meets requirements, a bottleneck may occur at some other points in the current large-scale infrastructure. For example, traditional tape-based backup solutions have higher latency than disks. It takes longer to access data from tape libraries than from disks. Although in the Large Hadron Collider (LHC) project disks are used as a staging area in front of the tape libraries in order to improve performance, this approach makes the system much more complicated. Moreover, the cost of disks has reduced over the years. Therefore, it is more efficient to use streamed replication of the original data. This enables multiple copies of the data to be stored at multiple locations for extra data protection.

A good data management system can provide extra protection for the data. It helps with tracking replications of the data and provides a recovery mechanism if the data are damaged. A good data management system may, in the future, provide features to federate data when collaborating with other institutes as well.

## Methods

### Data Grids

Many different science fields currently require dealing with large and geographically distributed data sets. The size of these data sets has been scaled up from terabytes to petabytes. For example, in high energy physics the LHC project at CERN produces several petabytes of data per annum [7]. In computational genomics, the WTSI generated more than 9 petabytes of data in 2010. In climate modelling, a large number of observed data, such as satellite images and simulation results, are regularly generated.

The combination of several issues, such as large datasets, distributed data and computationally intensive analysis, makes data management in a computational environment extremely complicated. There are several tools that have been developed to solve individual issues. However, a unified environment which allows users to deal with all these issues together is required. This environment is the so-called "data grid" [8].

In a computational environment, data may be stored in different locations with different storage devices. Applications should not need to be aware of these specific low-level data access mechanisms. In a computing grid, users should not worry about all the different local batching systems. With a single command, a job can be submitted to the computing grid and allocated to local computing resources. Similarly, in a data grid, applications should be presented with a uniform mechanism for accessing data.

The two fundamental services of a data grid are data access and metadata access. Data access services provide mechanisms to access, manage and initiate third-party data transfer in distributed storage systems. Metadata access services provide mechanisms to access and manage information about the data stored in storage systems. Several higher-layer data grid services have been developed on top of these two core services, such as data replication management and data filtering.

### The Storage Resources Broker (SRB)

The SRB, developed by the San Diego Supercomputer Center (SDSC), is a widely-used data grid solution. It has been adopted in many projects. The SRB provides a uniform client interface to different storage or file systems. Users can use the SRB to access distributed data from any network-accessible point. The SRB provides a virtual file system, with access to data being based on data attributes and logical names, rather than on physical locations or real names. The physical location is seen as a file characteristic only. One of the features of the SRB is that it allows users to easily replicate data across different physical file systems in order to provide an additional level of file protection [9].

The SRB also provides a metadata catalogue (MCAT) to describe and locate data within the storage systems. Files are actually stored in multiple "vaults". These vaults are repositories from which the MCAT server, which is used for mapping the location of logical and physical files [9], can extract files on demand.

### Integrated Rule-Oriented Data Systems (iRODS)

iRODS is an open source project developed by the Data Intensive Cyber Environments (DICE) research group as a successor to SRB but with significantly enhanced functionalities. The most important feature of iRODS is the Rule Engine. This allows data to be managed with policies, expressed as computer actionable rules. The Rule Engine can interpret these rules and perform a series of actions using microservices, which are a number of well-defined C language functions with a standard interface [10,11].

Rules can be invoked by users using a command line or iRODS API. However, rules automatically executed at policy enforcement points are more useful in our first stage use case, which mainly focuses on sequencing data preservation. Later stages will use iRODS for managing experiment outputs (such as alignment, annotation, etc.). Rules can be defined in multiple rule databases called *irb *files. The Rule Engine can interpret these files and perform the executions defined in *irb *files automatically.

The major difference between the SRB and iRODS is that the SRB needs to use "proxy" (remote server side) commands to invoke executables on the SRB server, whereas iRODS uses a simpler method which defines all the execution requirements in rules.

The most important feature of iRODS is its metadata function, which supports complex metadata defined by users. In order to minimize the load on the iRODS/SRB server, some other projects have decided to develop their own metadata systems to deal with metadata and to only let iRODS/SRB handle data access. For example, *e*Minerals developed the Rcommands server as a metadata server which allows users to extract and query data on their own [12]. However, Rcommands only provides key-value pairs. Users need to use an extra dictionary file to define the unit information. iRODS provides a triplet (key, value, unit) format to allow users to define their own metadata more comprehensively and store all the information within the same database instead of using another dictionary file. It should be noted that the iRODS metadata system does not contain semantic knowledge of the units at this moment. For example, given a value of 1,000 which uses grams as the unit, users can not query 1 kilogram instead. All values need to be converted to a single unit before they are submitted to the metadata system.

## Results

### iRODS implementation in the WTSI

Different data management systems are used in different fields. For example, dCache and CERN Advanced STORage manager (CASTOR) are widely used for large experimental data management in high energy physics. In genomics, traditionally, there is no requirement to manage such large amounts of data. Most data have actually been either stored in a database or remained as flat files. However, since sequencing technology has improved rapidly and now produces large amounts of data, without a proper data management system it would be easy to lose track of them.

**Figure 1:**
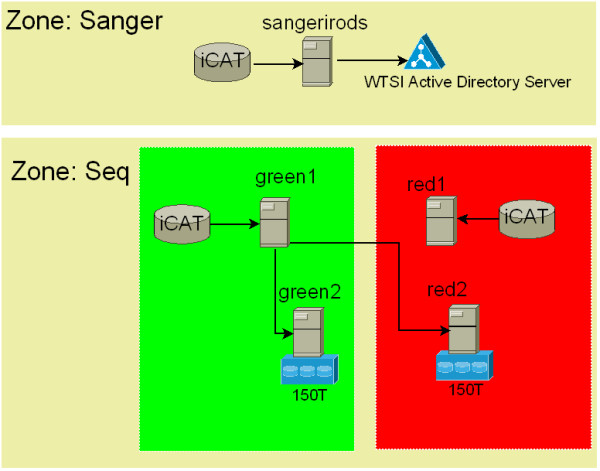
**irods-wtsi.png**. The architecture of the iRODS system in the WTSI. irods-sanger1 is the portal for authentication. green1 is the iCAT server. green2 and red2 are resources servers attached to the storage for extra data protection.

We have chosen iRODS as our data management system for the following reasons (Table 1): first, it is easy to deploy. iRODS has an installation script which helps system administrators to deploy it simply. It may take only 20 minutes to set up a basic system for use. Second, the metadata system helps users to find their data easily. A real-use scenario will be introduced in the next section. Third, the rule engine allows system administrators to define the rules and makes data replication and management much easier. Fourth, unlike dCache's mapping of a logical file name to a hash number based on a physical file name, iRODS can be configured to use the same name for both logical and physical file names. This is very useful at the first stage and enables system administrators to track files much more easily. However, with systems containing tens of millions of files alternate mappings are preferred (such as multiple levels of directory in-direction) to optimize the performance of the underlying file system. Figure 1 describes the architecture of the iRODS system implemented in the WTSI.

**Table 1:** Comparison of data grid solutions

	Deployment	Basic system set up	User metadata	Physical/logical file mapping
iRODS	easy	20 minutes	yes	Can be configured to be the same
dCache	medium	Few days	no	Hash number
CASTOR	hard	Few weeks	no	Hash number

As a first stage, the WTSI uses iRODS as a preservation system. The idea is to replicate and back up the data in different locations with extra protection. If one of the rooms in the data centre fails, users still can access the data from the server in another room. Moreover, the WTSI uses iRODS to manage users' metadata. The following section describes how deployment and configuration were carried out to achieve this goal.

The iRODS servers have been deployed in two physical rooms (green and red) in the WTSI data centre. We deployed an iCAT-Enabled Server (IES), logically named *green1*. This server is located in the green room. Two other iRODS servers without ICAT (IES), named *green2 *and *red2*, were connected to *green1. Green2 *is located in the green room with 150T SAN storage attached to it. *red2 *also has 150T SAN storage attached to it but is physically located in the red room. The other IES, called *red1*, is not actually used in daily operations. Currently, *red1 *is configured to point to the same Oracle iCAT database as *green1. Red1 *is a spare server for *green1*. If *green1 *goes wrong, *red1 *can be used as the main iCAT server. Oracle Real Application Clusters (RAC) could be implemented in the future to provide a more resilient iCAT.

In iRODS, "zone" means an iRODS system consisting of an iCAT-enabled server (IES), optional additional distributed iRODS servers (IRES) and clients. Each zone has a unique name. The first zone in the WTSI is called "*seq*", which refers to the sequencing zone. *green1*, *green2 *and *red2 *comprise the "*seq*" zone. All the data staged to the "*seq*" zone will be automatically replicated to both IRES. In order to achieve this, some simple rules are used. iRODS rules are defined using the following syntax:

actionDef|condition|workflow-chain|recovery-chain

*actionDef *is the identifier of the rule. *condition *defines when the rule will be invoked. *workflow-chain *is the sequence of actions that the rule will execute. *recovery-chain *is the sequence of actions that will be executed if the *workflow-chain *fails.

The following describes the rules used in our iRODS system. The first rule is used to define the default resource which users should use. In iRODS, a resource is a software/hardware system which stores data. For example, resource types can be the "Unix file system", "HPSS" or "Amazon S3". Currently, only the Unix file system is used in our local resources. In the future, we could use Amazon S3 as another resource if genomic data are released for public use or for backup in the cloud.

There are two resources in the "*seq*" zone, called *res-g2 *and *res-r2*; these two resources compose a resource group called "*seq*". There is a resource called *wtsiusers *in the "*Sanger*" zone. "seq" and "Sanger" are federated, so users can see data from both zones. When users log in to the WTSI iRODS system, they all use the *wtsiusers *resource by default. The *wtsiusers *resource in the "*Sanger*" zone only provides limited storage for users' home directories. We like users to use this zone mainly as a landing area. In order to access sequencing data, all users need to access the "*seq*" zone.

In order to access the data in the "*seq*" zone, zone federation has to be implemented. iRODS supports zone federation, which allows the information from the iCAT of each zone to be shared. With federation, users can see the data from each zone by simply using the iRODS unix commands *ils *and *iget *to access it.

The first rule shown below defines the default resource. It forces the "*seq*" resource group to be the default resource. All data coming in to the "*seq*" zone have to use the "*seq*" resource. There is no condition in this rule, meaning that it applies to all scenarios. "Nop" (no operation) means no recovery chain has been defined.

acSetRescSchemeForCreate||msiSetDefaultResc(seq, forced)|nop

Although the first rule forces users to use the "*seq*" resource group, the prioritisation of resources within the resource groups needs to be defined as well. The second rule defines the preferred resource as the "*seq*" resource group. In addition, it also mentions avoiding the use of *res-r2*. The idea is to use *res-r2 *as a backup resource. All the data will stage into *res-g2 *and then replicate to *res-r2*. Users are not allowed to write data into *res-r2 *directly. However, in recovery-chain, if *res-g2 *cannot be accessed users are still able to get data from *res-r2*. ## is the separator used to separate the micro services or rules in iRODS policy.

acPreprocForDataOjOpen||msiSetDataObjPreferredResc(seq%res-g2)##msiSetDataObjAvoidResc(res-r2)|msiSetDataObjPreferredResc(seq%res-r2)##nop

The third rule is straightforward. After the data has been put into the iRODS system the data object will be replicated to all the resources within the "*seq*" resource group.

acPostProcForPut||msiSysReplDataObj(seq, all)|nop

With the above three modified rules, all data staged into the "*seq*" zone will be automatically replicated to all the resources. The last rule is to switch off the trashcan function.

acTrashPolicy||msiNoTrashCan|nop

With the trashcan function enabled, even when data are removed from iRODS they are still physically located in the storage device. This will lead to an accumulation of redundant data in the disks. Thus, this function should be switched off. This policy could also be configured to periodically empty the trashcan in order to avoid decreasing system responsiveness with deletion requests.

The "*Sanger*" zone, with the server name *sangerirods*, is configured as a portal and used by everyone in the WTSI. The WTSI has its own directory services using LDAP and Microsoft Active Directory (AD). Ideally, it would be good if iRODS could support authentication against the existing WTSI LDAP server. In reality, iRODS only supports three authentication mechanisms: the iRODS password, the Grid Security Infrastructure (GSI) and Kerberos. In order to integrate the existing WTSI authentication system, the easiest approach at this moment is to make Kerberos work with the WTSI Active Directory server. If the GSI is used, certificates need to be generated and this creates extra work. The iRODS password and Kerberos have thus been chosen as the major authentication mechanisms for our iRODS system.

### WTSI Use Case

Currently, WTSI users are mainly using iRODS for managing and accessing sequencing Binary Alignment/Map (BAM) files, which are binary representations of the Sequence Alignment/Map (SAM) file format and keep the same information [13]. Each BAM file normally has an index file, called BAM Indexing (BAI). Users then use these BAM files as input as part of their applications. For example, the cancer genome pipeline uses icommands as function calls in the Perl module to access BAM files from iRODS for further analysis and research.

The new sequencing technologies group is responsible for the quality control of sequencing data and the uploading of data to iRODS. This group is managed by a project administrator account named "*srpipe*". This account has the privilege of writing and uploading sequencing data (BAM files) to the "*seq*" zone. All other users can only read files which have been released for public access.

The sequencing data generated from the sequencers were located in an NFS file system at the first stage. The existing sequencing pipeline has a tracking database, which stores information about data types and how the data were generated. Some Perl modules have been developed to obtain metadata by querying the database and staging files to the iRODS using *iput*, that is, the icommand for staging files to the iRODS system, and adding metadata by using *imeta*,, that is, the icommand for adding metadata.

Different metadata have been added to both BAM and BAI files. Currently, the following metadata are available for BAM files: **study**, **library**, **sample**, **id_run**, **lane**, **tag**, **tag_index **and **human_split**. id_run, lane and tag_index are all unique within the WTSI. Some BAM files don't have a tag_index, which means the file is for the whole run lane.

Some BAM files have non-consensual human data. These data contain human genomic information and are not supposed to be accessed by the public. Therefore, we split the files into two parts: human and non-human. The public is not normally able to see the human part. **human_split **(changing to alignment_filter) is used to indicate this situation.

Each BAM file belongs to one study, has one or more samples and forms one actual library for sequencing, and each sequence may have a tag sequence with it. Therefore, we added these metadata to the file: study, sample (one or more), library and an optional tag sequence.

Sequences in BAM may be aligned to a reference. Therefore the metadata 'alignment' has been created to indicate this. If there is an alignment, a metadata reference is added to indicate which one was used. The following are some examples.

Listing 1: BAM file with tag

imeta ls -d/seq/5635/5635_3#2.bam

AVUs defined for dataObj/seq/5635/5635_3#2.bam:

attribute: type

value: bam

attribute: lane

value: 3

attribute: sample

value: SZ0002

attribute: reference

value:

/nfs/repository/d0031/references/Streptococcus_equi/4047/all/bwa/S-equi-4047.fasta

attribute: study

value: Streptococcus equi genome diversity

attribute: tag

value: CGATGTTT

attribute: library

value: SZ0002 1560825

attribute: id_run

value: 5635

attribute: tag_index

value: 2

attribute: alignment

value: 1

Listing 2: BAM index file (BAI)

imeta ls -d/seq/5635/5635_3#2.bai

AVUs defined for dataObj/seq/5635/5635_3#2.bai:

attribute: type

value: bai

Listing 3: BAM without tag and only the human non-consensual part

imeta ls -d/seq/5261/5261_5_human.bam

AVUs defined for dataObj/seq/5261/5261_5_human.bam:

attribute: type

value: bam

attribute: study

value: Plasmodium falciparum Illumina sequencing R&D 1

attribute: reference

value:

/nfs/repository/d0031/references/Homo_sapiens/1000Genomes/all/bwa/human_g1k_v37.fasta

attribute: sample

value: PK0039

attribute: human_split

value: human

attribute: lane

value: 5

attribute: library

value: PK0039 455682

attribute: id_run

value: 5261

attribute: alignment

value: 1

WTSI users can thus query the metadata and access data based on their requirements. For example, searching data by study name:

Listing 4: Searching data by study name

imeta qu -z seq -d study = 'Hyperplastic Polyposis'

collection:/seq/5208

dataObj: 5208_2.bam

collection:/seq/5208

dataObj: 5208_3.bam

collection:/seq/5208

dataObj: 5208_5.bam

collection:/seq/5230

dataObj: 5230_1.bam

collection:/seq/5230

dataObj: 5230_2.bam

Currently, most BAM files are generated by HiSeq 2000; however, other BAM files converted from fastq are also inserted into iRODS. We are also using iRODS to test a fourth-generation sequencer from PacBio in terms of managing its data, which are mainly in the HDF5 format.

### WTSI Experiences

iRODS has been running as a production system at the WTSI for about half a year now. During this period, the three major user groups have provided us with their experiences and feedback. These groups are data management/system management staff, researchers adding data to the iRODS and researchers using/accessing data from iRODS.

The experience of the data management staff is that iRODS is generally working well. Although there have been some problems, such as network failures and bugs from a specific iRODS release, these problems have been fixed by replacing the hardware and installing iRODS patches.

In order to check whether the replications of each resource are identical, md5 has been inserted into both iCAT (this is done by using the -K argument in *iput*) and the metadata service (this is done with *imeta*, using an MD5 checksum and its value as one of the metadata parameters). A script has been developed and runs in the background to check whether the md5 values are consistent in both resources. If the md5 values are not identical in both resources, the script then checks the md5 in the metadata and finds out which resource holds the correct replica.

Around 20 out of 40,000 files have been diagnosed with different md5 values [Additional file 1]. In order to check whether the physical copies were actually broken, *md5sum *was executed on the local disk against those 20 logical files and their replications (40 files in total). Interestingly, only 5 of them were actual broken files, meaning that they returned different md5s from both resources. The md5s of the other 15 files were identical. This result indicates that the md5s in iCAT are incorrect. It seems that using *ichksum *or *iput -k *for checking md5s on iRODS may potentially return incorrect results, but this rarely happens.

The feedback from those researchers who add data to iRODS indicates that iRODS is currently performing well. However, they did encounter some problems with loading the data at the beginning. The jobs became stuck at the loading stage for a long time and did not return any error messages. The same amount of data sometimes took a long time and was sometimes very fast. It was fine to run 10-20 loading jobs at the same time but things slowed down when more jobs than that were run. After six months the system is now becoming stable. It seems that updating to iRODS 2.4.1 and installing patches fixed the problems.

The feedback from those users mainly accessing data from iRODS is very positive. Unlike in some other institutes, where users have limited computing knowledge, WTSI users have very strong bioinformatics backgrounds and can pick up new IT technologies easily. Users can use iRODS quite well by simply following the tutorial on the WTSI internal wiki.

Moreover, the WTSI has a standard procedure when evaluating a new system. A test system has been set up for different user groups, such as sequencing informatics, UK10K, 1000 genomics and the cancer genomic. The system team then asks for users' opinions and feedback after a trial period. In our case, most groups thought that iRODS could potentially meet their requirements and that its performance was quite good. We were then asked by the head of sequencing informatics to set iRODS up as a production system. Thus, the implementation and usage of iRODS are user-/scientist-driven, which reduces the amount of effort needed to promote the system to end users. These users also think that *iget *is quite useful because it can pipe the output to stdout.

## Discussion

There are some biological projects and institutes where iRODS has been used. Firstly, there is iPlant, a US project to develop a cyberinfrastructure to address a series of challenges in plant science [14]. Secondly, there is the Swedish UPPNEX project for providing computing and storage resources for NGS research. Thirdly, there is the Broad Institute, which is the MIT and Harvard genomic centre. Fourthly, there is the Genome Biology Unit at the University of Helsinki, and lastly the National Center for Microscopy and Imaging Research (NCMIR) is in charge of biomedical research imagery at UCSD. However, none of these actually provide/publish iRODS deployment details and use cases. This paper, in contrast, provides useful information about the iRODS system as implemented in the WTSI and some initial use cases.

A production iRODS system has been implemented and used at the WTSI. Apart from some hardware failures, the iRODS system has worked smoothly for the first six months of production. Some lessons have been learnt during the first stage of production.

Using Kerberos as the authentication system works; WTSI users can use *kinit *(obtain and cache a Kerberos ticket) and icommands directly as long as they have an account in the AD. However, Kerberos did not work perfectly for those users needing to access data from iRODS and execute jobs in a computing farm. The main reason for this is that Kerberos has a limited credential life time; if jobs are queued in the batching system, which is LSF in our case, the Kerberos credential may run out of validation time. This means that Kerberos needs to be able to support a non-interactive computing environment.

Theoretically, the valid life time can be configured by the AD administrator. Practically, it would go against WTSI policy to configure the credentials with an unlimited life time. This problem could be solved by implementing other services, such as AUKS [15] or a MyProxy server [16]. This approach, however, creates more work for administrators and extra learning for users. For example, users then need to learn how to upload credentials to the proxy server using different commands which they are not used to.

The easiest approach is to simply use the iRODS password system. When using *iinit *(store password so other icommands can be executed), the token is stored in the user's home directory, which is shared by NFS across the whole WTSI computing system. Most importantly, *iinit *does not have a limited life time and will be revoked only when *iexit *(to remove the token generated by *iinit *from the disk) is used.

Although using *iinit *works, it is not a perfect solution since two authentication systems must be used. One is the original AD and the other is the iCAT-enabled iRODS server which manages the user's information. These two systems cannot be synchronized and thus extra load for managing users' accounts is needed. We are currently using a script to register users for iRODS. It would be good if iRODS could be integrated with LDAP in the future, as discussed in the iRODS user group meeting, 2011 [17].

More and more projects within the WTSI have raised their requirements when using iRODS for data management. We use the iRODS zone federation approach to maintain flexibility when more projects join. The "*Sanger*" zone can be seen as a portal which manages all users. Currently, there is only one project zone, called "*seq*". Future zones could be called "*Cancer*", referring to the cancer genome project, or "*UK10K*", referring to the UK10K project. These zones would allow each project to design its own style and flexibly manage their own data.

At this moment, iRODS is only used to manage metadata related to BAM files. However, for scientists who are doing work downstream (such as alignment or annotation) of the pipeline, iRODS can be also used to improve scientific data analysis. The iRODS metadata system can be used to manage experiment outputs and users can query the metadata and produce tables or graphs for further analysis [18,19]. Different research groups at the WTSI are currently investigating this aspect of iRODS usage on various internal testbeds.

We are also planning to federate research data with other institutes using iRODS. The next step is to federate malaria data with Oxford University and some public data with the Broad Institute. Another important step could be federating with EBI, allowing data to be replicated directly to EGA.

## Authors' contributions

GTC carried out research into iRODS and the implementation of the iRODS system in the WTSI. GTC and PC designed the iRODS system architecture. GQ defined the metadata for genomic data management. KS and GTC worked on the implementation of Kerberos with iRODS. GC is the team leader coordinating the data management project.

## Supplementary Material

Additional file 1 **Appendix I: Broken File Checking**. Click here for file
