# Supplementary material for: Implementing a genomic data management system using iRODS in the Wellcome Trust Sanger Institute
Source: BMC Bioinformatics. 2011 Sep 9;12:361. doi: 10.1186/1471-2105-12-361 (PMC3228552; doi:10.1186/1471-2105-12-361)
Supplement: Additional file 1 — Appendix I: Broken File Checking. [file 1471-2105-12-361-S1.DOC]

Appendix I: Broken File Checking

| File name | iCAT md5 for green2 | | iCAT md5 for red2 | local md5 at green2 | local md5 at red2 |
| --- | --- | --- | --- | --- | --- |
| /seq/3703/3703_1.bam | | 12a70351e1d590ae1c396928c80484f0 | 1195bcc87bc3cb24adf9cd948990d750 | 12a70351e1d590ae1c396928c80484f0 | 12a70351e1d590ae1c396928c80484f0 |
| /seq/3873/3873_8.bam | | 2f32d287f494b26e599139e563a41fd4 | 7013b488b35fa51c3b4427ad1049904f | 2f32d287f494b26e599139e563a41fd4 | 2f32d287f494b26e599139e563a41fd4 |
| /seq/4714/4714_8.bam | | a42533d19871cc5052b7bc33408b7082 | 62cc46f6cd189ed6e7f2c827b69f061b | a42533d19871cc5052b7bc33408b7082 | a42533d19871cc5052b7bc33408b7082 |
| /seq/5349/5349_1#0.bam | | e9758c40510c96c10ccac04f65b1053b | 4bcd26d8a8821216c56e8f868366b639 | e9758c40510c96c10ccac04f65b1053b | 4bcd26d8a8821216c56e8f868366b639 |
| /seq/5350/5350_7#11.bam | | 0831a9a69e5f546060e653309bded93f | aeb1048977dfc925a71acfd896d18d9d | 0831a9a69e5f546060e653309bded93f | aeb1048977dfc925a71acfd896d18d9d |
| /seq/5364/5364_8#4.bam | | b17f3fc6c3c1beedff3c2940b0de4e61 | 58d0846de9cef5264b559e2ea70ce055 | b17f3fc6c3c1beedff3c2940b0de4e61 | 58d0846de9cef5264b559e2ea70ce055 |
| /seq/5453/5453_2.bam | | 48b60d3000924475391c3dbe04af80f1 | 7e96157ad945af2b02a53c6a04f2ffce | 48b60d3000924475391c3dbe04af80f1 | 7e96157ad945af2b02a53c6a04f2ffce |
| /seq/5753/5753_6.bam | | 973ff854de5ebd953a1bcfb16b6d8021 | 73856c2d03e1c81dff2aa762a98d6123 | 973ff854de5ebd953a1bcfb16b6d8021 | 973ff854de5ebd953a1bcfb16b6d8021 |
| /seq/5809/5809_4.bam | | aae7c6b1638131f02e94c5b1eb3ed6f6 | 46acfa106e9eaf9bf7839cda25e324fd | aae7c6b1638131f02e94c5b1eb3ed6f6 | 6f14dc41a7e45a30db4ede31305da2ae |
| /seq/5878/5878_8.bam | | a768aec4f6813f5f325473edde8e4fd0 | 8a96e9787ae2083e271e372c59018bc7 | a768aec4f6813f5f325473edde8e4fd0 | a768aec4f6813f5f325473edde8e4fd0 |
| /seq/5894/5894_5.bam | | 5c783a49d8d867a2a7d271c1cf2dc47b | 1afca5d6874f5c198c2416a571667000 | 5c783a49d8d867a2a7d271c1cf2dc47b | 5c783a49d8d867a2a7d271c1cf2dc47b |
| /seq/5910/5910_2.bam | | 1b50da4cbd08669c236e4aa646b9f44d | 3636215855c2f65d9f23e485be6ee4c2 | 1b50da4cbd08669c236e4aa646b9f44d | 1b50da4cbd08669c236e4aa646b9f44d |
| /seq/5964/5964_2#7.bam | | a00e5ad0acd40beba626a65b6657602a | b0ba02d387d90674be9be232aa864ba5 | a00e5ad0acd40beba626a65b6657602a | a00e5ad0acd40beba626a65b6657602a |
| /seq/6025/6025_5#7.bam | | b8096fd1ec937bfc44150733e748b5ee | 23287cfd08abd1f57597405888a0c5ae | b8096fd1ec937bfc44150733e748b5ee | b8096fd1ec937bfc44150733e748b5ee |
| /seq/6052/6052_6.bam | | 3f8d05859619ada917403207023a9914 | 84ec08b5d4ce6201d1630109ff07fcb3 | 3f8d05859619ada917403207023a9914 | 3f8d05859619ada917403207023a9914 |
| /seq/6074/6074_6.bam | | 98480114df878da2aa594dd088efd805 | 508fb73cfd9e7cb68c4e1bcce719804f | 98480114df878da2aa594dd088efd805 | 98480114df878da2aa594dd088efd805 |
| /seq/6153/6153_8.bam | | 5623b38ae16f953b5e7f75f798788358 | a7f49b93a972edbdceb473a708e9854a | 5623b38ae16f953b5e7f75f798788358 | 5623b38ae16f953b5e7f75f798788358 |
| /seq/6207/6207_6#4.bam | | b485bf090ff54916bec94c2b7c783ca4 | e6cca84d3fd275a48633deb78650f8ae | b485bf090ff54916bec94c2b7c783ca4 | b485bf090ff54916bec94c2b7c783ca4 |
| /seq/6221/6221_3#7.bam | | 1866814edaf440133257fc901ff037b0 | f6e52accc207a148f58a4fc49d1f7541 | 1866814edaf440133257fc901ff037b0 | 1866814edaf440133257fc901ff037b0 |
